# *Cyprinus carpio* Decoction Improves Nutrition and Immunity and Reduces Proteinuria through Nephrin and CD2AP Expressions in Rats with Adriamycin-Induced Nephropathy

**DOI:** 10.1155/2012/237482

**Published:** 2012-05-20

**Authors:** Yumei Qi, Huijuan Xiao, Changjie Xu, Xiaojian Tian, Hui Wu, Wei Shen

**Affiliations:** ^1^Department of Nutrition, Tianjin Third Central Hospital, Tianjin 300170, China; ^2^Tianjin Key Laboratory of Artificial Cells, Tianjin Third Central Hospital, Tianjin 300170, China; ^3^Department of Nutrition, Second Affiliated Hospital of Tianjin University of Traditional Chinese Medicine, Tianjin 300000, China; ^4^Institute of Integrative medicine, Qingdao University Medical College, Qingdao 266021, China; ^5^Department of Nutrition, The First Affiliated Hospital of Soochow University, Jiangsu, Suzhou 215000, China

## Abstract

*Cyprinus carpio* decoction (CCD) is a well-known Chinese food medicine formula, accepted widely as a useful therapy in preventing edema and proteinuria caused by renal disease. However, the mechanism underlying this effect remains unclear. The current study investigated the potential mechanism of CCD in alleviating nephropathy induced by adriamycin (ADR) in rats. 70  eight-week-old *Wistar* rats were randomly divided into normal, model, fosinopril, YD, YG groups. All rats except for the normal group received 6.5 mg/kg·bw of ADR injection into the vena caudalis once. Different doses of CCD (11.3 and 22.5 g kg^−1^) were lavaged to rats in YD and YG groups, respectively. Then the serum biochemical values of the total protein (TP), albumin (ALB), blood urea nitrogen (BUN), creatinine (Cr), electrolyte levels, and the urinary protein (UP) content in 12 hr urine were measured. Interleukin-4 (IL-4) and interferon (INF-**γ**) were measured by enzyme-like immunosorbent assay (ELISA). The pathomorphological analysis was observed using light and electron microscopy, and the expressions of nephrin and CD2-associated protein (CD2AP) in renal tissues were determined by immunohistochemical assay. The results indicated that CCD can relieve ADR-induced nephropathy (ADN) by improving the nutrition status, regulating the immunity, and inhibiting proteinuria by increasing nephrin and CD2AP expressions.

## 1. Introduction

The prevalence of chronic kidney disease (CKD) is on the rise in all ethnic groups. It is a major global issue with serious harm to human health, characterized by proteinuria, hypoalbuminemia, edema, hypertension, and abnormal renal function that progresses to end-stage renal disease (ESRD) finally [1]. Adriamycin- (ADR-) induced nephropathy (ADN) in the rats is usually used for many studies on the mechanism of various therapies [2, 3]. The common denominator of this experimental nephropathy, which mimics minimal-change disease, is a toxin-induced noninflammatory glomerular foot process effacement that evolves to focal segmental glomerulosclerosis (FSGS) and tubulointerstitial injury resulting in nephrotic syndrome (NS) [4]. It was shown that there was a close relationship between NS and immune dysfunction of T helper cells (Th): Th1 and Th2 cells. The findings suggest that the immune imbalance with dominant Th2 cells, which causes the abnormal levels of interferon-gamma (INF-*γ*) mainly secreted by Th1 cells and interleukin-4 (IL-4) by Th2 cells, contributes to the glomerular disease through promoting proteinuria [5]. Proteinuria is one of the important risk factors, as well as for the performance in the development of progressive renal disease [6, 7]. It is well established that podocytes play an important role in maintaining the integrity of the glomerular filtration barrier [8]. The discovery and identification of nephrin, a transmembrane slit diaphragm protein, have improved our understanding of the molecular mechanisms involved in the maintenance and function of the glomerular filtration barrier and its role in the pathogenesis of clinical and experimental proteinuria [8]. Recently, several additional components of the slit diaphragm have been identified, including CD2-associated protein (CD2AP), which interacts with the cytosolic tail of nephrin [9]. Disruptions in nephrin and CD2AP have been identified as the cause of several inherited and acquired forms of proteinuria [8, 9].

*Cyprinus carpio* decoction is a well-known Chinese food medicine formula. As written in the Compendium of Materia Medica by Li, it is characterized by sweet and gentle nature and surprising activities of alleviating water retention, strengthening the spleen, and eliminating dampness, with channel tropism to spleen, lung, kidney, and liver [10]. Body water metabolism is correlated with the function of lung, spleen, and kidney in the traditional chinese medicine (TCM) theory. Based on the theory, it is also believed that CCD can invigorate the spleen, and replenish lung qi, as well as regulate water passage [10, 11]. It is a useful therapy for edema caused by serial diseases, including nephropathy. It has been extensively used for improving edema, ascites, pleural effusion induced by nephropathy, liver, cardiac disease, cancer or pregnancy and polyhydramnios in China for centuries. It had been ever documented in TCM classics, for example, the Essential Recipes for Emergent Use Worth A Thousand Gold written by Simiao Sun in Tang Dynasty [12], the Medical Secrets of an Official by Tao Wang in Tang Dynasty [13], and the Taiping Prescription of Holy Benevolence by Huaiyin Wang in Song Dynasty [14]. In modern clinical therapy, it is often treated in refractory edema and receiving unbelievable efficacy. Professor Guan [15] and Li [16] treated nephropathy with severe proteinuria and edema in a prescription of CCD. They found its alleviating action was significant and it made those symptoms difficult to recur. However, the mechanism is unclear in modern western medicine. In the nutrition view,* Cyprinus* is digestible and rich in protein, microelements and vitamins, for example, A, B1, C. The ample free amino acid makes it very delicious. It is a good source of fat acid, myristic acid, palmitic acid, oleic acid, linoleic acid, and linolenic acid, especially EPA and DHA. Although almost each component has its function, such as antioxidation of the decomposed products from the fresh [17] and vitamins A and C, there is no finding in which one or some molecules combined play the role in inducing diuresis to alleviate edema yet. It is still a mystery and needs further study. 

Although blockade of the rennin-angiotensin-aldosterone system (RAAS), either by angiotensin-converting enzyme or angiotensin II (AT II) AT-1 receptor blockers, has been shown to be useful in reducing proteinuria and controlling renal damage in nephrotic and nonnephrotic renal diseases, its clinical efficacy is not necessarily satisfactory [18–20]. However, administration of *Cyprinus carpio* decoction is increasingly adopted in clinic and civil, which receives surprising efficacy. Investigations have showed that *Cyprinus carpio* decoction has the capacity to prevent urinary protein in rats with ADN [21, 22]. Our previous work also testified this effect. Nevertheless, little is known about the mechanism by which *Cyprinus carpio* decoction alleviates ADN. Therefore, we aimed to investigate here how the administration of *Cyprinus carpio* decoction could play a protective effect in rats with ADN.

## 2. Methods

### 2.1. Induction of Nephropathy in Wistar Rats

 70 eight-week-old male *Wistar* rats (Shandong Lukang Pharmaceutical Co. Ltd. of China), weighing 160~200 g, were randomly divided into five groups: normal group (N, 10 rats), adriamycin-induced nephrosis model group (M, 15 rats), fosinopril group (F, 15 rats), and therapeutic groups with low and high dose of CCD (YD and YG, 15 rats in each group). All rats were kept and cared under condition that the temperature indoor was 23 ± 2°C and the relative humidity 50~70. They were maintained on standard forage and water *ad libitum* throughout the experiment. Nephropathy was induced by administration of adriamycin according to a method previously described [23] after one-week acclimation. Briefly, each rat except N group was injected into vena caudlis with 6.5 mg adriamycin (Zhejiang Hisun Pharmaceutical Co. Ltd., China) kg^−1^ body weight once. All experiments were performed in accordance with the NIH's *Guiding Principles in the Care and Use of Laboratory Animals*.

### 2.2. CCD Preparation

Huanghe common carp, *Cyprinus carpio haematopterus *Temminck et Schlegel (Henan Academy of Fishery Science, China), was chose to prepare *CCD*. Frozen immediately after harvest and thaw it at room temperature before preparation. Weigh *Cyprinus carpio *and put them into a stainless steel pot after washing them in cold water three times. Then add to the pot distilled water, which is four times the weight of the carps. After boiling 10 min, stir the mixture and make the flesh of fish separated from the bone. Continue heating the mixture gently and stir it once every 10 min until its total weight was four times as much as that of the fish, which was about 60 min. Next, close fire and filter out *Cyprinus carpio *decoction (25% concentration) by a 6-drug screen (100 mesh). Make it condensed to the concentration of 400%, which is 4 g carp per milliliter, with rapid vacuum concentrator (type 5301, Eppendorf China, Ltd.) at 45°C. Split it and pack with polyethylene plastic bags at 100 mL/bag. Disinfect them 60 min in hot water at 80°C after sealing. Make the decoction cool naturally, and store them in −20°C refrigerator.

### 2.3. Products/Drugs Tested Fed to the Treatment Groups of Rats

 Fosinopril is a kind of angiotensin-converting enzyme inhibitor, which is commonly used to treat chronic renal disease. At week 3, rats in fosinopril (Sino-American Shanghai Squibb Pharmaceuticals Ltd., China) group were fed as positive control by oral gavage at a dosage of 10 mL fosinopril solution (0.09 mg/mL)kg^−1^ body weight. The required amount of fosinopril was made up with physiologic saline and then force-fed to the rats with a strainless steel stomach tube. YD and YG group were fed via oral gavage with 10 mL CCD kg^−1^ body weight at 1.13 g and 2.25 g *Cyprinus carpio* per milliliter, respectively. Normal and model rats received an identical volume of vehicle. Normal chow was provided to all rats. Feeding of fosinopril, CCD, and vehicle were discontinued after the experimental period of nine weeks.

### 2.4. Common Condition Monitoring

 During the experiment, record the weight each week and chow consumption each day.

### 2.5. Serum, Urine, and Kidney Tissue Samples Preparation

At the end of each week, 12 hr urine samples were obtained from each rat. When the experiment was over, obtain serum samples from rats. Then, all rats were sacrificed under general anesthesia using i.p. injection of chloral hydrate. Kidneys were removed immediately for histological examinations.

### 2.6. Clinical Parameter Measurement

Serum levels of total protein (TP), albumin (ALB), urea nitrogen (BUN), creatinine (Cr), sodium, potassium, and chloride were determined with commercially available kits (Ningbo Rui Bio-technology Co., Ltd., China). Urinary protein (UP) excretion in 12 hr was also detected with a commercial kits (Leadman Group Co., Ltd., China). These serum and urine parameters were measured by automatic biochemistry analyzer (Olympus AU640, Olympus Optical Co., Ltd., Japan).

### 2.7. ELISA Assay for Cytokines

 Interleukin-4 (IL-4) and interferon (INF-*γ*) were measured by enzyme-liked immunosorbent assay (ELISA) (RT-6000, Rayto, USA) as described in the user's manual.

### 2.8. Morphometric Analysis

 Kidney sections were fixed in 10% neutral-buffered formalin, blocked in paraffin wax, and stained with hematoxylin-eosin (H&E) for light microscopic analysis. For electron microscopy, small blocks of the kidneys were fixed in 4% glutaraldehyde, postfixed in 2% osmium tetroxide, dehydrated in graded ethanol, and then embedded in epoxy resin. Ultrathin sections were stained with uranyl acetate and lead citrate.

### 2.9. Immunohistochemical Assay

For immunohistochemical analysis of CD2-associated protein (CD2AP) and nephrin, kidney sections were fixed in 4% paraformaldehyde, embedded, and sliced routinely. The sections (4 *μ*m) were immersed in citrate buffer solution (0.01 M, pH 6.0) and heated at 120°C in autoclaved sterilizer for 10 min, naturally cooled for 30 min, and then immersed in 3% aqueous hydrogen peroxide (H_2_O_2_) for endogenous peroxidase ablation at room temperature for 30 min. The following steps were executed in a moist chamber according to the procedure described in the manual of kits, CD2AP and nephrin antibody (Beijing Biosynthesis Biotechnology Co., LTD, China). Finally, the tissue sections were counterstained with hematoxylin, dehydrated, cleared, and mounted with neutral gums. In parallel, tissue specimens in which the primary antibody was replaced by PBS served as negative control. Specificity was established by demonstrating the loss of immunoreactivity in matched tissue sections. The positive signal of CD2AP and nephrin proteins was brown or yellow granular mass that could be used to trace and measure the aforementioned proteins. The positive signals of these proteins were measured by two pathologists using Image-Pro Plus version 6.0 Image Analysis System (Media Cybernetics, Inc., USA). Five fields with more than 10 glomeruli in each fields per section were chosen randomly and analyzed. The average optical density (AOD) which represented the positive staining intensity was calculated as the ratio between the integrated optical density and the sum area with positive cells in analyzed fields.

### 2.10. Statistical Analyses

All data are presented as means ± SD. Repeated measures ANOVA and one-way ANOVA analysis were performed for comparison in different groups by using SPSS 17.0 software. Differences were considered significant when *P* values were <0.05.

## 3. Results

### 3.1. Diet Consumption and Rats Weight

**Figure 1:**
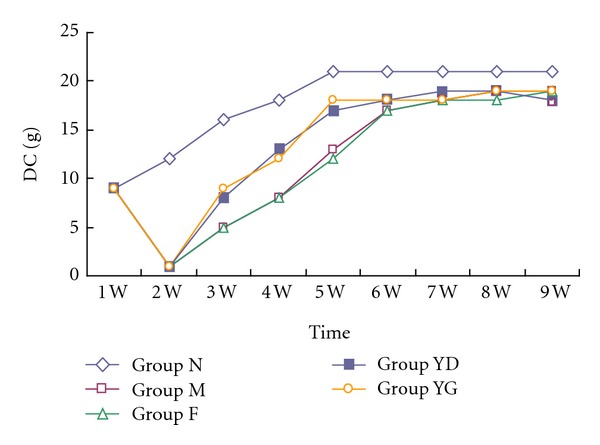
Diet consumption (DC) in the five groups. All values are expressed in mean ± SD.

**Figure 2:**
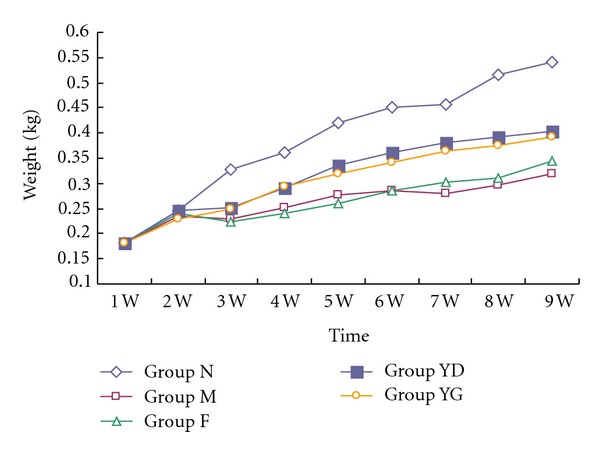
Weight in the five groups. All values are expressed in mean ± SD.

At week 3, rats in YD and YG groups eat 8 and 9 g diet per day, respectively. It was more than M and F groups (5 g) (*P* < 0.05). The consumption gradually increased in every group from week 1 to week 5. However, there was no difference in each rat (*P* > 0.05) afterwards (see Figure 1). They ate about 20 g chow one day. Rats grew with time. At the end of the third week, the weights of rats in the experimental groups were 0.230 ± 0.029, 0.225 ± 0.027,0.252 ± 0.045, and  0.249 ± 0.017 kg. They began lower than normal group (0.328 ± 0.023 kg) (*P* < 0.05). At the next week, it was occurred that significant differences existed during four experimental groups (*P* < 0.05). However, there was no statistically differences between YD and YG groups (*P* > 0.05), as shown in Figure 2.

### 3.2. Light Microscopic Observation

**Figure 3:**
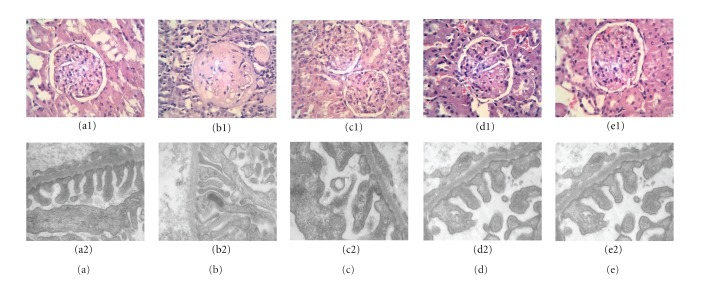
Histopathological analysis of rats' kidney sections by light microscopy (Row 1) and electron microscopy (Row 2). (a) Normal group (N), (b) model group (M), (c) fosinopril group (F), (d) YD group (YD), and (e) YG group (YG). Magnification, ×400.

It showed that, in the rat kidney of M group, there were obvious pathological lesions characterized by glomerular swelling, stenosis of the renal glomerulus capsular space, blurry margin between each part, many inflammatory cells around glomerulus, and abundant protein exudation in the renal tubular lumen (Figure 3(b1)). In the rats' kidney of F, YD, and YG groups, these pathological damages were lighter than those of M group, although glomerular swelling, inflammatory cells around glomerulus, and protein cast in the renal tubular lumen were also sporadically observed. The severity of pathological damage was reduced (Figures 3(c1), 3(d1), and 3(e1)). No pathological changes were found in the kidney of control group (Figure 3(a1)).

### 3.3. Electron Microscopy Observation

The ultrastructural changes of rat kidney in the five groups were comparatively analyzed by electron microscopy. In control group, there were no apparent damages as judged by intactness of renal glomerulus of rats' kidney tissue. Clear foot process was observed on the visceral surface of renal glomerular epithelial cells, which was no swelling, hypertrophy, and inflammatory cells around them (Figure 3(a2)). In M group, the basal structure of renal glomerulus was damaged. Foot process diffusely changed, became thin and flat, and fused, even disappeared. The line of glomerular basement membrane (GBM) was unclear, and there were a lot of inflammatory cells in renal interstitial cells (Figure 3(b2)). In F, YD, and YG groups, the lesions were significantly relieved, although foot process effacement and inflammatory cells were observed in the renal glomerulus (Figures 3(c2), 3(d2), and 3(e2)). There was no difference in electron microscopic morphology in these three groups.

### 3.4. Clinical Parameters

As shown in Table 1, administration of high dose of CCD significantly prevented the decrease in serum albumin and total protein (*P* < 0.05, versus group M). There was no significant difference in serum BUN, Cr, Na^+^, K^+^, and Cl^−^ concentration among each group (*P* > 0.05). At the third week after induction, the rats excreted plenty of protein in urine, 76.78 ± 8.63, 69.66 ± 15.29, 77.49 ± 12.83, and 88.25 ± 15.05 mg. Urinary protein excretion in 12 hr urine of the rats received fosinopril, and CCD was greatly decreased (*P* < 0.05), compared with ADN rats, although it was higher than that of the normal rats (*P* < 0.05).

### 3.5. Cytokine Level

**Figure 5:**
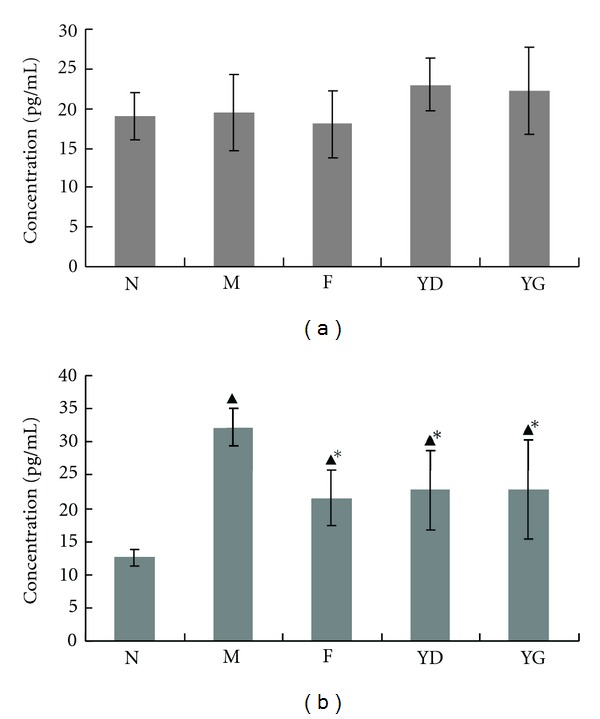
levels of serum interferon-gamma (INF-*γ*, (a)) and interleukin-4 (IL-4, (b)). Data are shown as means ± SD. ^▴^ *P* < 0.05 versus group N, *P < 0.05 versus group M.

As shown in Figure 5, CCD can increase the amount of INF-*γ* production, although the difference was not statistically significant. The level of IL-4 in model group (32.13 ± 2.84 pg/mL) was greatly higher than that in normal group (12.55 ± 1.21 pg/mL). Fosinopril and CCD can dramatically decrease the level of IL-4, when compared to the model group. However, there was no significant difference among groups F, YD, and YG. It was shown that CCD was as effective as the fosinopril.

### 3.6. Nephrin and CD2AP Expression

**Figure 6:**
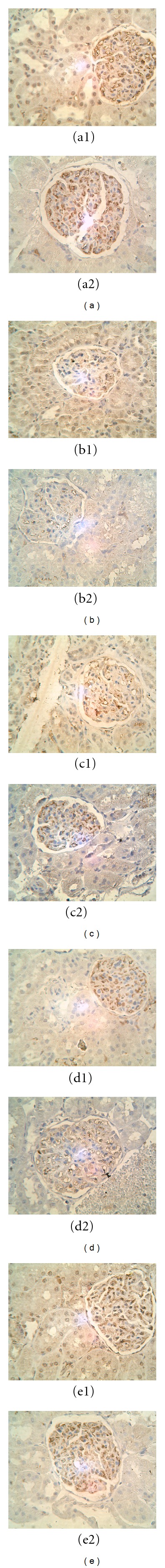
Expression of CD2AP (Row 1) and nephrin (Row 2) in each group. (a) Normal group (N), (b) model group (M), (c) fosinopril group (F), (d) YD group (YD), and (e) YG group (YG). Magnification, ×400.

**Figure 7:**
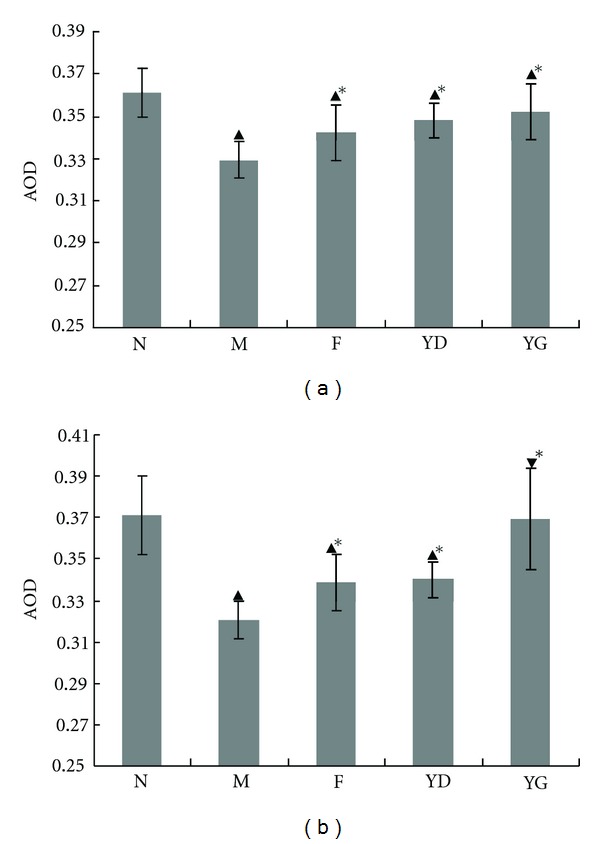
The average optical density (AOD) of CD2AP (a) and nephrin (b) expression in rats' kidneys in the five groups. Data are shown as means ± SD. ^▴^ *P* < 0.05 versus group N, *P < 0.05 versus group M, ^▾^ *P* < 0.05 versus group F.

 Brown or yellow granular mass, in a continuous shape of thick line, was found in the glomerular capillary wall, the attachment of podocytes, in each group. The expressions of nephrin and CD2AP in renal tissues of ADNs were decreased with comparison of that in normal group, especially the model group the lowest. Its positive expressive area was distributed in punctuation or irregular clumps. In the rats received Fosinopril and CCD, the area distribution was more than that in group M (Figure 6). Semiquantitative analysis of staining intensity showed that the expressions of nephrin and CD2AP decreased in groups M, F, YD, and YG, compared with the normal group. However, it had some degree increase in the three treatment groups (*P* < 0.05, versus group M). See Figure 7.

## 4. Discussion

Protein-energy malnutrition (PEM) is common in patients with chronic kidney disease (CKD) and worsens as the CKD progresses toward the end-stage renal disease (ESRD). This condition is one of major predictors of poor clinical outcome in kidney failure. The data suggested that improving nutritional status may have a better impact on overall health-related quality of life in CKD [24, 25]. In our study, food consumption and body weight were dramatically decreased in the rats of AND from the third to the fifth week. It may be caused by metabolic changes of protein in tissue and plasma with anabolism increase and catabolism reduction [26]. Malnutrition in nephropathy made the rats eat less forage due to bad appetite, which further aggravated the weight loss. CCD is a well-known traditional Chinese medicine formula made up of* Cyprinus carpio, *a kind of fish, which is rich in protein and microelements and can be cooked to be a delicious food. Rats in groups YD and YG that received CCD ate more food than the model rats and those treated with fosinopril from the third week to the fifth week, although all rats had similar consumption at the end of experiment. Consequently, it prevented the weight reduction. In addition, administration of this food medicine also enhanced the levels of total protein and albumin. It may be the result of protein anabolism enhancement and proteinuria reduction induced by CCD. These findings suggest that CCD can improve anorexia and malnutrition induced by nephropathy.

Several immunological abnormalities have been associated with NS, especially helper T lymphocyte function. There are two kinds of helper T cells; one is T helper-1 (Th1) lymphocyte, secreting primarily INF-*γ*. The other is T helper-2 (Th2), producing mainly IL-4. The activity of Th1 and Th2 cells was defined by the production of INF-*γ* and IL-4. Their serum concentration change can reflect the immunity balance status [27, 28]. It was demonstrated in several studies that INF-*γ* played a toxic effect on kidney by breaking up intercellular tight junction of glomerular visceral epithelial cells and promoting foot process effacement [27, 29]. However, there is no consensus concerning the production of INF-*γ* in patients with NS. Wang et al. [30] reported that the INF-*γ* level in the active stage of NS was significantly greater in comparison with normal children, and it decreased in remission stage. But Stachowski et al. [31] reported that it was lower in children with NS and grew up to the level of healthy children in the remission. In this study, there was no difference in INF-*γ* level among the five groups. It may be associated with the remission at the end of experiment, when the serum samples were prepared. The toxicity of IL-4 on kidney had been described in previous experiments [27, 32]. It induced adhesion molecule expression by combining with IL-4 receptor in glomerular visceral epithelial cells and directly affected the cell structure and permeability, leading to proteinuria. It was tested that there was a positive correlation between IL-4 and urinary protein [32]. We observed that rats administered with CCD produced less IL-4 than those in ADN. The effect was the same with fosinopril. It may be indicated that CCD can reverse the immune imbalance with dominant Th2 cells by reducing the secretion of IL-4 and improve the immune function, which is helpful to relieve ADN.

Proteinuria is an important risk factor for the progression and prognosis of chronic renal disease [33, 34]. Therefore, the pathophysiologic mechanisms underlying its development and improvement under several therapies are focused on by many researchers [35–39]. The slit diaphragm, bridging the space between adjacent foot processes, was assumed to make an important contribution to the molecular sieve for glomerular filtration. Recently, increased insight in the molecular makeup of the podocyte foot processes and slit diaphragm, including nephrin and CD2AP, led to the support for the pivotal role of these structures in the maintenance of permselectivity [35, 36].

Nephrin is a member of the immunoglobulin superfamily of transmembrane cell adhesion molecules [37]. It is localized to the slit pore of podocytes and contributes to the integrity of the SD. Several investigators have examined the integrity of the slit diaphragm in acquired proteinuric diseases, using the slit diaphragm protein nephrin as a marker. It had been reported that there was no nephrin and SD disappeared under electron microscopy in the patients with inherited nephrotic syndrome [38]. It has been recently found that nephrin expression level is reduced in experimental animal models and human-acquired renal disease with proteinuria [39, 40]. Nephrin gene knock-out mice died of severe proteinuria shortly after birth. Benigni et al. [41] reported that the glomeruli of rats with massive proteinuria showed a progressive decline in nephrin mRNA and protein content from 1 wk to 8 mo after disease induction. These studies suggest that nephrin may play an important role in the development of proteinuria.

CD2AP is expressed primarily in glomerular podocytes at the cytoplasmic face of the SD domain where it interacts with nephrin and podocin [9, 42]. Lack of the glomerular expression of CD2AP in animals produces mesangial cell proliferation with extracellular matrix deposition, glomerulosclerosis, and extensive foot-process effacements [43] that are correlated with the entity of the defect. Shih et al. [43] reported that mice with CD2AP gene knockout died of renal failure progressed from massive proteinuria shortly after birth and observed the damaged podocytes and foot process fusion and effacement, like the pathological change in nephrotic syndrome. Intervening on CD2AP may be the pathophysiological basis developing the proteinuria in renal disease. In our study, proteinuria relieved after using CCD in the rats with ADN, as well as glomeruli inflammation and foot process effacement reduced. It can be explained by the increment of nephrin and CD2AP expression. Thus, it is established that nephrin and CD2AP are critical in maintaining the integrity of the slit diaphragm and preventing proteinuria.

In conclusion, the present study shows that CCD has a protective effect against ADR-induced nephropathy, and this effect may be attributed to the improvement of nutrition status and regulation of cytokines and immune balance. Moreover, the roles of CCD are related to the reduction of proteinuria through promoting nephrin and CD2AP expressions. The findings may shed light on the pharmacological basis for the clinical application of the traditional Chinese medicine in the treatment of chronic kidney disease. However, which materials play the pharmacological activities, some free amino acids rich in CCD or others? It is still unclear. Thus, it needs further study on the acting compounds of CCD.

## Figures and Tables

**Figure 4:**
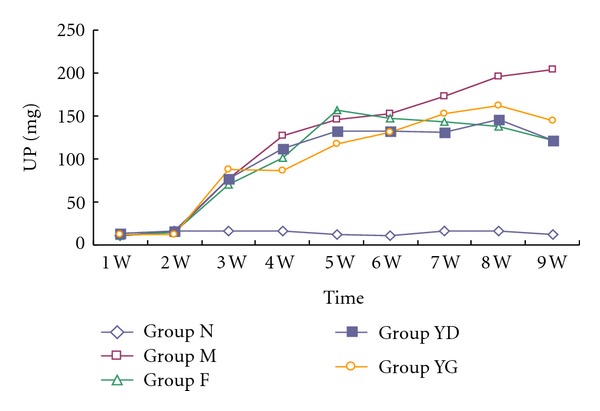
Urinary protein (UP) excretion in 12 hr urine in the five groups. All values are expressed in mean ± SD. At the end of experiment, UP is 12.42 ± 4.63 mg in the normal group (N), 204.15 ± 48.84 mg in the model group (M), 121.16 ± 32.80 mg in fosinopril group, 122.29 ± 15.00 in the group with low dose of CCD (YD), and 144.25 ± 16.65 mg in the group with high dose of CCD (YG). The latter three groups had less UP than the model group. However, there was no difference among them.

**Table 1:** Changes in serum parameters in the five groups.

Group	TP (g/L)	Alb (g/L)	BUN (mmol/L)	Cr (*μ*mol/L)	Na^+^(mmol/L)	K^+^(mmol/L)	Cl^−^(mmol/L)
Group N	68.29 ± 4.29	23.18 ± 0.97	0.09 ± 0.01	79.43 ± 7.51	146.00 ± 1.41	6.25 ± 1.45	102.10 ± 0.99
Group M	59.37 ± 6.28^▴^	11.13 ± 2.05^▴^	0.16 ± 0.07^▴^	73.99 ± 7.81	147.67 ± 1.63	6.62 ± 0.93	102.17 ± 4.40
Group F	66.51 ± 7.76*	12.74 ± 3.83^▴^	0.12 ± 0.03	75.06 ± 13.44	143.57 ± 3.51	8.08 ± 0.90^▴∗^	100.43 ± 2.94
Group YD	67.10 ± 4.79*	14.18 ± 4.00^▴^	0.12 ± 0.04	77.55 ± 11.13	147.13 ± 4.64	7.66 ± 1.84^▴^	101.38 ± 3.20
Group YG	68.95 ± 4.97*	16.45 ± 3.99^▴∗▾^	0.10 ± 0.03	83.43 ± 11.14	151.50 ± 2.07^▴∗▾^	6.92 ± 0.55	105.38 ± 3.62^▴▾^

TP, total protein; Alb, albumin; BUN, blood urea nitrogen; Cr, creatinine; Na^+^, sodium ion; K^+^, potassium ion; Cl^−^, chloride ion. All values are expressed in mean ± SD. ^▴^ *P* < 0.05 versus group N, *P < 0.05 versus group M, ^▾^ *P* < 0.05 versus group F.
